# *Ms Radiance*: A community-led social media mHealth pilot for transgender women

**DOI:** 10.1080/26895269.2025.2503224

**Published:** 2025-05-13

**Authors:** Lahari A. Telang, Hezreen Shaik Daud, Aoife G. Cotter, Abdul Rashid

**Affiliations:** a RCSI-UCD Campus Malaysia (RUMC), Penang, Malaysia; b School of Medicine, University College Dublin, Dublin, Ireland; c Centre for Experimental Host Pathogen Research (CEPHR), Dublin, Ireland; d Volunteer Penang Family Health Development Association, Malaysia, Individual with Lived Experience, Penang, Malaysia

**Keywords:** ADDIE instructional design, dental services utilization, mHealth, oral transmission of STIs, patient and participant involvement and engagement (PPIE), transgender women

## Abstract

**Background:**

Transgender women in Malaysia face marginalization and experience barriers to accessing healthcare. Formative research conducted by the research team has identified low utilization of dental services, alongside gaps in knowledge and a perceived low risk of STI transmission through oral sexual practices. Tailored educational interventions that address the specific needs of transgender women in Malaysia are essential for bridging these gaps.

**Methods:**

A focus group discussion (FGD) was conducted with patient and public involvement and engagement (PPIE) advisors, and the use of a social media-based mHealth educational intervention to create awareness was discussed. The ADDIE (Analyze, Design, Develop, Implement, and Evaluate) instructional design was employed alongside the transgender community involvement in designing and developing the “Ms. Radiance” intervention. PPIE was crucial for presenting the educational material as artistic bilingual posts, stories, and reels that were peer-reviewed and culturally sensitive. The feasibility and acceptability of the intervention were assessed among transgender women (*n* = 30) through a three-week pilot launch on Instagram. An online pretest/post-test questionnaire was utilized to evaluate the intervention’s impact on condom use for oral sex, oral hygiene practices, and dental service utilization.

**Results:**

The themes derived from the FGD were *Content of the Educational Intervention, Social media as a mode of delivery, Community engagement* and *Sustainability of the intervention.* Ms. Radiance demonstrated feasibility, achieving 100% retention and a 93% post-intervention completion rate. It was considered acceptable, as shown by positive post-intervention feedback, with 79% of participants indicating they would recommend the intervention to others.

**Conclusion:**

A first-of-its-kind intervention for transgender women in Malaysia was designed and developed with PPIE and pilot-tested. The pilot’s feedback will help improve the intervention for a scaled-up version of the study.

## Introduction

The global response to sexually transmitted infections (STIs) has faced challenges in recent years, leading to a plateauing of disease burden (Elendu et al., 2024; World Health Organization). To this effect, the World Health Organization (WHO) has emphasized the need for people-centered and integrated services to aid in achieving universal health coverage (UHC) as a strategy to impact efforts toward ending human immunodeficiency virus (HIV) infection and sexually transmitted infections (STIs) by 2030 (World Health Organization, 2022). Creating tailored actions for specific vulnerable populations is essential for the successful implementation of these efforts (Kuhns et al., 2021). Strategies that integrate patient and public involvement and engagement (PPIE) through co-design and participation at multiple levels in the process have been shown to achieve better health outcomes (Louise & Annette, 2019). PPIE in health research incorporates participants’ lived experiences to ensure the research is relevant, acceptable, and beneficial. The involvement of participants, including patients, in the research process can range from consultation and advisory roles to more integrated approaches (Brett et al., 2014; Ocloo et al., 2021).

In Malaysia, transgender women are considered disadvantaged, often overlooked, and marginalized (Gibson et al., 2016). Changing gender assigned at birth is subjected to criminalization by both civil and religious laws of the country, making it difficult for transgender people to express their identities freely (Afiqah, Rashid, & Iguchi, 2022; Draman & Hashi, 2019; Haniff et al., 2021; Teh, 2002). Stigma and discrimination undermine essential determinants of health, including social security, education, employment, family support, and access to healthcare (Tan, Lee, & Cheong, 2021; Zay Hta et al., 2021). A complex interplay of social, legal, and individual factors leads to marginalization, which compounds the barriers to accessing and receiving healthcare (Afiqah et al., 2022; Telang et al., 2025). Malaysia is among the few countries in Asia that have achieved UHC, which ensures that all citizens have equal access to healthcare, including oral healthcare (Rannan-Eliya et al., 2016). However, healthcare in general may be neglected by transgender women due to the harsh context in which they operate, prioritizing survival over healthcare, which may seem secondary given their struggles with stigma, family rejection, and violence (Gibson et al., 2016; Rashid & Afiqah, 2023; Rutledge et al., 2018).

To optimize healthcare equality in marginalized populations, it is essential that public health strategies reach them through innovative and new methods (Ginaldi & De Martinis, 2024). One such approach is the use of social media. There is strong evidence of the effectiveness of mobile health (*mHealth*) interventions for promoting health through the use of social media (Escobar-Viera et al., 2021). With the inexorable increase in digitization, leading to seamless internet access worldwide, utilizing mHealth interventions through social media is crucial for reaching marginalized populations, whether intentionally or by choice (Escobar-Viera et al., 2021). This is particularly helpful for sensitive topics such as sexual health and STIs (Gabarron & Wynn, 2016). Malaysia is a developing upper-middle-income country in Southeast Asia, boasting a robust cellular network and extensive internet penetration (Department of Statistics Malaysia Official Portal). A recent study on Malaysian adults’ views of *mHealth* found that awareness of *mHealth* applications was higher among younger generations, and participants were open to change and willing to use technology to improve their health (Lee, Wong, & Lee, 2020). Moreover, there is evidence to show the acceptability of *mHealth* interventions among transgender women in Malaysia and the need to focus on such interventions to promote sexual health and reduce the transmission of STIs (Gautam et al., 2024; Krishnan et al., 2021). Social media have been reported to promote the well-being of gender-diverse people through the improvement of social networking (Berger et al., 2022; van Hierden, Dietrich, & Rundle-Thiele, 2021). That being said, social media as a potential health educational tool deserves active research among closely knit yet marginalized communities, particularly transgender women, owing to the disparities in prevention and health care that prevail (Berger et al., 2022; Cao et al., 2017; Escobar-Viera et al., 2021). Hence, the need for a tailor-made *mHealth* educational initiative to address knowledge gaps was deemed essential, along with the efficient use of social media to deliver this mHealth initiative to reach the target population effectively.

To conduct research with marginalized groups, building trust was an essential step in improving commitment from both the transgender community and the researchers (Spinuzzi, 2005). Formative research was conducted by our team of researchers in 2023 to identify the needs of the transgender community to get a better understanding of their lived experiences (Telang et al., 2025). Barriers and enablers of dental service utilization and safe oral sexual practices were explored through in-depth interviews with a group of transgender women from Northern Malaysia and focus group discussion (FGD). The barriers to dental service utilization were not noted to be specific to the transgender community but rather deeply rooted in the cultural belief that dental treatment is painful and visits to the dentist were associated with fear and anxiety (Telang et al., 2025). Esthetics was prioritized over the health of the oral cavity, leading many to seek nonprofessional dental care from beauticians. Oral sex, primarily receptive fellatio with or without ejaculation with a cis-male client or partner, was routinely practiced by most transgender women who participated in the study (Telang et al., 2025). The majority (80%) perceived the risk of transmission of STIs to be low through oral sexual practices when compared to anal or genital sex and hence did not see the need to use condoms for their clients or partners (Telang et al., 2025). These preliminary results, along with the awareness of the existing gap in dental service utilization (Tan et al., 2021), prompted the need for an educational intervention for the transgender community.

The importance of collaborative partnerships and community-engaging strategies in sexual and gender minority research has been emphasized more than ever in the recent decade (Bonett et al., 2023). Co-designing enables end-users to be involved in various aspects of the research process, acting as consultants or advisors (Sánchez de la Guía, Puyuelo Cazorla, & de-Miguel-Molina, 2017). Therefore, the aim of this narrative is to describe the design and development of an educational intervention to improve dental service utilization and promote safe oral sexual practices in relation to the transmission of STIs for transgender women in Malaysia. PPIE was integral to the project, ensuring the participatory design and development of a bilingual, peer-reviewed, and culturally sensitive social media-based educational intervention. End-user experiences, feasibility, and acceptability of the intervention were assessed through feedback obtained from a pilot study. The results of the pilot would inform the future planned scaled-up version of the study, which would assess the overall impact of the educational intervention.

## Methodology

The present study is part of a larger project aiming to improve oral and sexual health promotion for transgender women in Malaysia. Ethics approval was obtained from the local ethics authority of the Joint Ethics Committee of the School of Pharmaceutical Sciences, USM-Hospital Lam Wah Ee on Clinical Studies (USM-HLWE/IEC/2023 (0003)) as well as the Human Research Ethics Committee of University College Dublin (UCD) Human Subject (Sciences) (LS-23-68-Telang-Cotter).

### Reflexivity and positionality

The authors of this article include cisgender and transgender individuals. A reflexive approach was maintained throughout the study period through respectful communication, ensuring confidentiality, valuing each other’s feedback, and transparent reporting. Transgender women were directly involved in participant recruitment, data collection, analysis, and sharing of findings. Three of the four authors are from the Global South, with two of them being Malaysian. One of the authors is a transgender woman who received training in PPIE research methodology and the remaining authors have experience working with gender-diverse populations. All authors involved in the interpretation of data acknowledge their identities and experiences may influence the interpretation and are committed to ensuring a reflexive approach free of potential biases.

The study was divided into two interconnected phases. Phase 1 involved the design and development of the educational intervention, as well as pilot testing. This would be followed by phase 2, which is the scaled-up version of the study (not described in this report). The design and development phase, which spanned nearly two years, built trust and earned the confidence of the transgender community, paving the way for the project (Telang et al., 2025). What started as a collaboration between the principal author (LAT, a cisgender woman) and the research assistant (HSD, a transgender woman activist) expanded to include local transgender artists and other community members, ultimately forming a PPIE advisors’ team. PPIE played a key role in the conceptualization and design of the study. The involvement of transgender community members was crucial to ensure the cultural sensitivity of the educational intervention. Research objectives were outlined, and topics that would define the content of the educational intervention were conceived. Formative analysis of the topics was conducted during the FGD with a mixed group of PPIE advisors. The authors have used the ADDIE (Analyze, Design, Develop, Implement, and Evaluate) instructional design (Rossett, 1987) to describe the methods section.

#### Analysis

A focus group discussion (FGD) was conducted with PPIE advisors, who provided key insights into the educational intervention. These advisors were carefully selected from a pool of individuals who had lived experiences and/or were working in the field of transgender healthcare. Among the participants (*n* = 7) who consented were four transgender women, two of whom were local community advocates, one transgender activist, and one artist. The other three participants were cis-identified and included a public health physician from the local “friendly clinic” (clinics receiving a higher volume of HIV and STI cases), a psychological counselor specializing in gender and sexual minorities’ matters, and a general dentist working for a local non-governmental organization (NGO) for women’s welfare and vulnerable groups. The discussion was audio recorded and transcribed verbatim. The data were transcribed and analyzed using NVivo 12 Windows software. The transcripts were reviewed by the research team members multiple times to enhance the reliability of the findings. A multistep coding process was employed, where auto-generated codes were compared with manually generated ones to ensure consistency and accuracy. Investigators reached a consensus on the most relevant ideas, which were then coded and grouped into minor themes. Through thematic analysis, these minor themes were further refined into major themes. Additionally, direct quotations from participants were recorded to capture their experiences, opinions, knowledge, and emotions. A thematic approach to data analysis (Braun & Clarke, 2006) facilitated the exploration of inductively generated results, which were underpinned by the Information, Motivation, and Behavioral Skills (IMB) theory (Fisher & Fisher, 1992). The notable use of the IMB theory in HIV and STIs has been linked to effectively promoting prevention and treatment behaviors (Fisher & Fisher, 1992; Wang et al., 2020).

The themes derived from this analysis laid the foundation for the design and development of the intervention. The suitability of a social media-based educational intervention to increase awareness was explored. The educational intervention was tailor-made to the needs of transgender women in Malaysia and peer-reviewed by their own community members. The design and development of the educational intervention were guided by the theoretical framework of the Health Belief Model (Champion & Skinner, 2008) and Andersen’s behavioral model of health services use (Andersen, 1995). The scope of educational intervention was to cover core topics that helped achieve the research objectives. The ADDIE instructional design was used to create a conceptual framework by mapping the content of the educational intervention with the research objectives (Telang et al., 2025). The engagement of transgender community partners through PPIE at various levels of the project was evaluated using the GRIPP2-SF checklist (Staniszewska et al., 2017).

#### Design and development

The process of developing the educational intervention took over nine months, commencing in March 2023 and concluding in December 2023. The aim was to prepare the content of the educational intervention by first listing key components identified through existing evidence-informed literature and merging them with the knowledge gaps identified through formative research (Telang et al., 2025). These topics were then aligned to the project by mapping them to the research objectives. Keeping in mind that the content must be culturally sensitive and tailored to the needs of transgender women in Malaysia, it was essential that the content undergo community review. The content was made bilingual, in English and Bahasa Malaysia. Back-and-forth translation into both languages, incorporating local slang words, was used to make the message more relatable to the audience. This was done with the help of local activists from the transgender community. The name “*Ms Radiance*” was also chosen by the PPIE advisors as it reflects happiness, beauty, and good health seen on one’s face (Cambridge Dictionary, 2024). Inputs from the PPIE advisors and consultations with experts in the field further strengthened the content. The content was then verified for scientific accuracy and relevance by three research team members, who are content experts in the fields of public health (AR), infectious diseases (AC), and oral medicine (LAT). Table 1 outlines the blueprint detailing the mapping of research objectives to educational topics, as well as the mode of delivery and assessment.

**Table 1.** Educational intervention mapping and blueprint.

Intervention/Research objectives	Topic themes	Mode of delivery	Mode of assessment
Change in condom use behavior for oral sexual practices	Oral diseases in relation to STIs Safe oral sex practices Frequent testing	Instagram posts, stories, and reels (static images, videos, or animations with captions and links to information)	Mixed methods-Questionnaires, interviews, FGD
2. Oral hygiene practices	Oral hygiene maintenance Effects of smoking and vaping		
3. Dental service utilization	Dental checkup and its importance Brushing techniques, choosing the right oral hygiene aids		

(STIs: sexually transmitted infection; FGD: focus group discussion).

The role of the artists was to simplify the content and convey the message through visual art. By incorporating positive transgender representation into the artwork, they helped make the content both appealing and ethically reflective (Caldwell, 2022) simultaneously (Figure 1). Fictitious animated transgender characters in the iconic pink and blue colors of the transgender flag were hand-drawn and then digitized. The art form of Cubism was also used to depict the artist’s impression of these characters (Ganteführer-Trier, 2004). Each of these posts were again vetted carefully for scientific accuracy, relevance and appropriate referencing by the research team.

**Figure 1.**
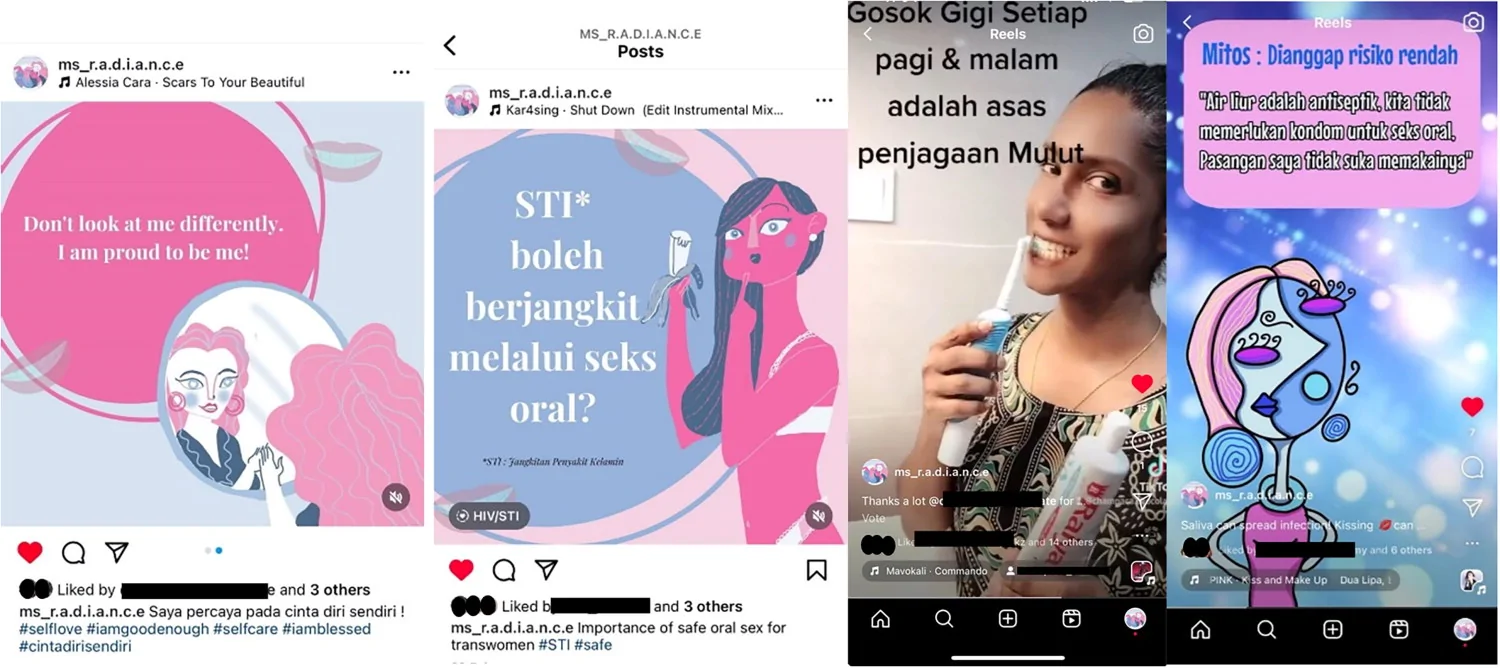
Sample post and reels from *Ms radiance* on instagram.

### Implementation and evaluation

#### Study setting for the pilot

Social media, particularly Instagram, was deemed suitable for implementing the intervention following discussions with the PPIE advisors and community members. Firstly, high internet penetration in Malaysia (Department of Statistics Malaysia, 2021) meant that the intervention would potentially have a wider reach to an otherwise hard-to-reach population. Secondly, Instagram can be a powerful platform for health education, as it emphasizes visuals with engagement-friendly features and has the potential to foster connections through social networking there building a community around health and wellness (Picazo-Sánchez, Domínguez-Martín, & García-Marín, 2022; Sharpe, 2024). Thirdly, utilizing an established social media platform such as Instagram for the intervention, rather than developing a standalone mobile application, would enhance the likelihood of engagement and visibility, given the platform’s substantial user base in Malaysia (Howe, 2024; Jerome, 2023).

#### Participants and recruitment

Participants were recruited through word of mouth and snowball sampling, assisted by PPIE advisors who leveraged their community networks and drew from the pool of individuals involved in the initial exploratory research. Inclusion criteria to participate were: any Malaysian person identifying as a transgender woman or in the transfeminine spectrum, eighteen years and above in age. The formally translated bilingual (Bahasa Malaysia and English) participant information sheet, consent form, and questionnaires were edited for inclusivity and cultural sensitivity by the authors in consultation with community members.

#### Pilot testing

The intervention through *Ms Radiance* was launched on Instagram for pilot testing for a period of three weeks. The pilot test was conducted as an online pretest/post-test mixed methods study with a single group of transgender women participants. The primary goal of the pilot was to assess the feasibility and accessibility of the educational intervention, while the secondary goal was to evaluate the outcomes in relation to the research objectives. With no similar published studies, we believe that this pilot study was the first of its kind among transgender women in northern Malaysia.

The participants who consented were directed to an online pre-intervention questionnaire. Upon successful completion, they were sent a link to join the Instagram group via WhatsApp, which included a quick response (QR) code for easy access. A separate mobile device and phone number with WhatsApp were used exclusively for the study, which was handled only by one of the authors (HSD). The Instagram account was private, created using the university email address (RCSI-UCD Malaysia Campus), and was managed exclusively by the principal investigator (LAT) to safeguard the data of group members. No additional data was collected through observation of interactions in the group, except for the number of likes and plays. Data privacy and security are critical elements in social media-based research (Berger et al., 2022). Hence, it was imperative that all community guidelines prescribed by the application provider be adhered to during the entire study period. Every effort was made to ensure that the content created for *Ms Radiance* complied with the Malaysian Communications and Multimedia Act 1998 guidelines, specifically excluding prohibited or offensive content (Section 211) and/or improper use of network services (Section 233) in Malaysia. To ensure a respectful environment throughout the intervention period, basic housekeeping guidelines were posted, comments were disabled, and daily account monitoring was conducted. During these 3 wk, the educational content was posted 2–3 times per week in the form of posts, stories, and reels. The topics posted during the pilot were oral diseases in relation to STIs, safe oral sex practices, frequent testing, oral hygiene maintenance, effects of smoking and vaping, dental checkups and their importance, brushing techniques, and the right oral hygiene aids. Participants could view the posts and stories at their convenience, and if they needed clarification, they could send a direct message to the Instagram handle.

The consent provided by participants ensured the feasibility and acceptability of the pretest. The feasibility of the intervention was determined by the participant retention rate and post-intervention test completion rate of over 80% at the end of 3 weeks of the pilot, based on findings from similar interventions (Aschbrenner et al., 2022; Lacombe-Duncan et al., 2020). The acceptability would be measured using qualitative data from the post-intervention open feedback provided by the participants and their chances of recommending it to other transgender women. This feedback would also be used to improve the intervention for the scaled-up phase of the study. The mean difference in pretest and post-test measures to assess the effectiveness of the intervention was directed toward the research objectives through self-reported items in the questionnaire. These were changes in condom use behavior for oral sexual practices, oral hygiene practices, and dental service utilization. Data analysis was done using IBM SPSS statistic software (version 29) (IBM Corp 2023). Descriptive statistics were reported in frequencies and percentages. However, the assessment of these self-reported items was not expected to lead to significant changes, given the short duration of the pilot, and hence only a brief comparison of pretest and post-test variables has been included.

## Results

This section describes the thematic analysis of FGD with PPIE advisors and the pilot study results.

### Thematic analysis

The themes derived from thematic analysis of the FGD with PPIE advisors were the *content of the educational intervention, social media as a mode of delivery, community engagement, and the sustainability of the intervention* (Table 2).

**Table 2.** Thematic analysis of FGD with PPIE advisors.

Major themes	Minor themes
Content of the educational intervention	Verified by experts Relevant and exclusive for transgender women Culturally sensitive content Fun and engaging Bilingual Easy to understand
Social media as a mode of delivery	Popular Easily accessible Already existing social network Privacy and confidentiality Participant retention
Community engagement	Peer reviewed Local artists from transgender community Positive influence from community members Foster sisterhood Constructive feedback
Sustainability of the intervention	Useful for future training and education Accessible to local non-governmental organizations (NGOs)/welfare organizations

(FGD: focus group discussion; PPIE: patient and public involvement and engagement).

### Content of the educational intervention

The PPIE advisors valued content reviewed by professionals to ensure credibility. They emphasized the importance of providing tailored information that addresses the unique health concerns of local transgender women. Sensitivity to Malaysian cultural and social contexts was a key requirement. Moreover, the content posted on social media must be fun and engaging. Preference for interactive, visually appealing content over text-heavy formats was suggested. The need for accessibility in both Bahasa Malaysia and English was frequently mentioned, underscoring the importance of creating bilingual content. Simplicity in language and presentation was considered essential for inclusivity, making it easy to understand.

The information must be curated for specific transgender needs for safe sexual health and oral health promotion. Of course, it should be easy to understand, with minimal medical jargon and complexity, so that viewers can benefit from it. R001, a cis-identified physician.

Through the content, we should make it obvious that we are talking about transgender issues… it beats the whole purpose if the information is too general. R002, a cis-identified counselor.

It should be 100% only about transgender women, an exclusive group just for us! (Exclaims with excitement).… Or should I say an ‘inclusive group’ for my sisters? (grins)….I would definitely want to be in it. R003, a transgender woman community advocate with lived experience.

Health is a topic that can be boring to discuss. The information must be both humorous and promote health at the same time – funny and light-hearted. R004, a transgender woman artist.

Transwomen are very cynical people; sometimes, our jokes can be harsh and serious… our lives are already too hard…. So, keep it simple and clean (referring to the content of the educational intervention) … if we don’t want followers to write nasty comments or, worse still, unfollow the group. R005, a transgender woman community advocate with lived experience.

### Social media as a mode of delivery

Most participants were already active on Instagram, making it a convenient choice. Social media provide free and instant access to information, and leveraging existing transgender community networks enhances outreach. Regular updates and interactive features (e.g. quizzes and live sessions) were suggested to keep users engaged. However, concerns about privacy, anonymity, and discretion in accessing information were raised. Suggestions for openly addressing ways to mitigate these concerns through an informed consent process were given.

We are already on Instagram daily, so it makes sense to use it for learning too. R005, a transgender woman community advocate with lived experience.

On social media, the pictures do the talking. Therefore, the artwork must convey the message with minimal text, key captions, and relevant hashtags. R004, a transgender woman artist.

Most transwomen in my community are on Facebook and Instagram. When compared to Facebook, Instagram is more appealing to the younger generation, particularly because it focuses on pictures and videos. R006, a transgender woman activist.

The account must be private, and participants must be invited to follow it. That way, only verified people will be allowed in—not a potential saboteur or a bot. (with a smirk on her face). R006, a transgender woman activist.

### Community engagement

Content validation by transgender individuals was suggested to ensure relevance and acceptability. Participants suggested using community members to create relatable visuals and videos. Role models from the transgender community can inspire engagement. A sense of solidarity and support within the platform was seen as crucial. Continued feedback mechanisms were suggested to improve the content quality.

A pre-launch survey should help gather feedback. It is more like a community opinion through a pilot that can help test uncharted waters. R007, a cis-identified dentist.

If our own community members are involved, the content will feel more personal and trustworthy. For example, a make-up video featuring one of our sisters who is giving health advice… legit! (meaning legitimate). R003, a transgender woman community advocate with lived experience.

I believe that if the content’s information is engaging, the platform will surely retain its followers. Young transgender women are more likely to be drawn to trending things. Too much information focusing on HIV is an overload. New information… lesser-known content… for example, dental veneers and the latest products that can improve your smile. These topics will work. (Nods head confidently) R006, a transgender woman activist.

### Sustainability of the intervention

Participants expressed interest in utilizing the content for long-term education. Collaboration with organizations was suggested to ensure sustainability beyond the research.

If NGOs and welfare groups support this initiative, it can continue helping new people in the community, R001 cis-identified physician

The benefit of this research should also focus on building social networking and support. So, it is important that the educational content is made accessible to welfare organizations working with transgender women. R002, a cis-identified counselor.

Based on the themes analyzed in the FGD, the educational intervention was designed and developed with community engagement, as described in the methodology. The pilot test results are described under the headings of implementation and evaluation phases of the ADDIE model.

#### Pilot test results

Thirty participants consented to participate in the pilot test. All of them identified as transgender women had a mean age of 41 (range, 26 to 59) years, and were residing in and around Penang state in northern Malaysia at the time of the study. Participant characteristics, as they relate to the outcomes of the pilot study, are presented in Table 3. Twelve (40%) participants had ever engaged in transactional sex, with 22 (73.3%) having multiple partners. Eighteen (60%) claimed never to have engaged in condom-less sex in a given month; 7 (23.3%) occasionally engaged in condom-less sex, and 5 (16.7%) were unsure. Eleven (36.7%) participants admitted their partners or clients who were cisgender males did not use condoms while they engaged in oral sex, either oro-anal or oro-genital (receptive fellatio with or without ejaculation).

**Table 3.** Characteristics of participants and sexual engagement.

Participant characteristics (*n* = 30)	*n* (%)
Ethnicity Malay	30 (100%)
Education level completed Primary school Secondary school Tertiary level	1 (3.3%) 20 (66.7%) 9 (30%)
Current sexual orientation Bisexual Gay/lesbian Heterosexual/Straight Pansexual Others*	1 (3.3%) 7 (23.3%) 13 (43.3%) 2 (6.7%) 7 (23.3%)
Sexual behavior: the sex of your partner/s Individuals of the same sex Individuals of different sex Multiple sexes	22 (73.3%) 7 (23.3%) 1 (3.3%)
Sexual attraction: Whom do you feel physically and emotionally attracted to? Men Both men and women	29 (96.7%) 1 (3.3%)
Engagement in sex work/transactional sex Yes No	12 (40%) 8 (60%)
Number of sexual partners in a month Single partner Multiple partners	8 (26.7%) 22 (73.3%)
Engagement in unprotected/condom-less sex (genital/anal) in a month Never Occasional Unsure	18 (60%) 7 (23.3%) 5 (16.7%)
Partner condom usage while engaging in oral sex, i.e. oro-anal or oro-genital (receptive fellatio with or without ejaculation) Yes No	19 (63.3%) 11 (36.7%)
Current HIV status Negative Negative, on PrEP Positive, on medication Not sure	23 (76.7%) 5 (16.7%) 1 (3.3%) 1 (3.3%)
Current STI status** Negative Not sure	23 (76.7%) 7 (23.3%)

(*Note*: Others* = some participants preferred not to specify any sexual orientation; **Refers to Syphilis/Gonorrhoea/Chlamydia/HPV/herpes)

HIV: human immunodeficiency virus; PrEP: preexposure prophylaxis; STI: sexually transmitted infections.

The retention rate of participants was 100% by the end of the 3 wk, with all of them following the Instagram group. The post-intervention questionnaire was completed by twenty-eight participants, resulting in a 93% completion rate. Hence, the pilot intervention was considered feasible. The most essential items assessed in the pretest/post-test questionnaires were condom usage (by male partner) during oral sex (receptive fellatio), oral hygiene practices immediately after oral sex, and dental service utilization (measured by scaling done). A McNemar test was conducted to determine whether there was a statistically significant change in condom usage by male partner during oral sex (receptive fellatio) before and after the intervention. The results indicated no significant difference between the pretest and post-test responses, *χ*^2^ (1, *N* = 28) = 0.000, *p* = 1.000 (Exact). Similarly, no significant difference was seen between oral hygiene practices immediately after oral sex *χ*^2^ (1, *N* = 28) = 0.000, *p* = 1.000 (Exact). A Wilcoxon Signed-Rank Test was used to assess the change in dental service utilization by assessing scaling done between the pretest and post-test. The results showed no statistically significant difference, *Z* = 0.722, *p* = 0.470, indicating that the 3-week pilot intervention did not produce a significant shift in reported utilization of dental services. The summary of the results is displayed in Table 4.

**Table 4.** Summary of pretest/post-test comparison.

Variable	Pretest (*n* = 30)	Post-test (*n* = 28)	Statistical test *χ*^2^/Z	Significance
Condom use (male partner) for oral sex (receptive fellatio) No Yes	14 16	12 16	*χ*^2^ = 0.000	*p* = 1.000 (Not significant)
Oral hygiene practice after oral sex No Yes	7 21	6 22	χ^2^ = 0.000	*p* = 1.000 (Not significant)
Dental service utilization (scaling done) Never Rarely/cannot remember Once a year or more often	3 12 15	3 11 14	*Z* = 0.722	*p* = 0.470 (Not significant)

*Note: χ*^2^ = McNemar test; *Z* = Wilcoxon signed rank test; McNemar test used exact significance due to small sample size. Wilcoxon test based on 28 paired observations (test statistic *W* = 23.000).

Feedback regarding the intervention and their overall experience was collected, and the results are displayed in Table 5. Most participants were highly satisfied (*n* = 23, 82%) with the educational intervention; 22 (79%) found the information provided regarding the health of transgender women to be both relevant and helpful in managing their health. Twenty-five (89%) participants found the information trustworthy, and 22 (79%) were likely to recommend it to others. The overall impression of improving oral health practices was rated as four stars by 10 (36%) and five stars by 13 (46%) of them. The intervention was considered acceptable based on this feedback and the chances of recommending the intervention to other transgender women. Instagram analytics could not be assessed, as the account was private. Additionally, the engagement of participants on social media could not be used as a measure of acceptability, as comments were turned off for privacy and security reasons. However, within the short timeframe of 3 wk, each of the posts received an average of 5-6 likes, with the brushing video by a transgender woman receiving the highest number of likes at 16, surpassing any of the other posts.

**Table 5.** Evaluating the educational intervention, *Ms radiance* on Instagram.

	Feedback (n = 28)	Very low/Low		Undecided/Unsure	High/Very high	
1.	How satisfied were you with the educational intervention, *Ms Radiance*, overall?	–		5 (18%)	23 (82%)	
2.	How relevant was the information provided by Ms. Radiance for the health of transgender women?	1 (4%)		5 (18%)	22 (79%)	
3.	How useful was the information provided by *Ms Radiance* in helping you manage your health?	1 (4%)		5 (18%)	22(79%)	
4.	How much do you trust the information provided by *Ms Radiance*?	1 (4%)		2 (7%)	25 (89%)	
5.	How likely are you to recommend *Ms Radiance* to other transgender women?	1 (4%)		5 (18%)	22 (79%)	
6	How do you rate the overall impression of *Ms Radiance* for improving oral health practices?	One star 2 (7%)	Two stars –	Three stars 3 (11%)	Four stars 10 (36%)	Five stars 13 (46%)

The responses to open-ended feedback were reviewed, and the consolidated summary of the written feedback was divided into two themes- what the participants liked about the intervention and the scope for improvement or critical suggestions for the future. Twenty-four of the 28 participants provided positive feedback describing the intervention as “informative,” “useful,” and “easy to understand.” The information provided was trans-friendly (e.g. “I liked the information about STIs for only transgender women”*).* Oral health information was appreciated (e.g. “I liked the oral health part, something we often overlook”). Suggestions for the future primarily focused on continuing the study with additional posts, particularly those related to oral health (e.g. “I would like to learn more about dental veneers”). The use of a more accepted colloquial term for transgender women, “Transnita” (Bahasa Malaysia), was suggested. Few of the participants felt that videos were better than static images. Critical yet valuable suggestions were made regarding the organization of the content in terms of sequence and the post-intervention questionnaire being too lengthy and time-consuming.

## Discussion

In this report, the authors describe the design and development of a *mHealth* educational intervention for transgender women in Malaysia, utilizing the ADDIE instructional design in the methods section. The role of PPIE at various steps of the development process has also been credited. The results describe the thematic analysis of the FGD with PPIE advisors, followed by self-reported variables in pretest/post-test analysis and the feasibility and acceptance of Ms. *Radiance* on Instagram.

To simplify the health education that the authors aimed to provide, it was important to reach the target population (transgender women of Malaysia) where they already are: on social media. Instagram is a popular social media platform in Malaysia, with a wide reach and high user engagement and retention (Howe, 2024). Instagram was utilized to its advantage for engaging participants in this research. Moreover, involving participants at an interpersonal level through social media-based sexual health interventions is one of the successful methods for bringing in targeted behavioral change (Condran, Gahagan, & Isfeld-Kiely, 2017). Existing social media platforms are more likely to foster long-term relationships with their users compared to standalone apps, thereby enhancing the overall retention and success of the intervention (Cao et al., 2017). The artists who contributed emphasized the creativity and visual appeal that resonated with the end user, a focus also reflected in comparable smartphone-based educational interventions (Hill & Johnson, 2020; Kuhns et al., 2021; Sun et al., 2020). The participants valued the emphasis on *Ms Radiance* being an exclusive social media group for transgender women. This finding aligns with the opinion expressed by transgender women who participated in testing a sexual health promotion mobile app developed in North America (Sun et al., 2020). In order to meet the specifics of identity representation and health information needs of transgender women in Malaysia, it was imperative to keep the group exclusive. Moreover, maintaining exclusivity also helps with understanding how the users engage with the platform (Escobar-Viera et al., 2021).

PPIE in this research project provided us with the potential to improve the awareness and relevance of the research within the given timeframe and resources. We employed a decentralized approach, as recommended in participatory design research (Spinuzzi, 2005), by first identifying the community’s needs and establishing trust. The impact of PPIE may be less well-documented in studies from the Global South (Cook et al., 2019), yet our collaborators were quick to adapt and understand their roles and what the project demanded of them. The benefits of community partnership facilitated access, participant recruitment as well as interpretation and contextualization of the results (Bouman, 2018; T’Sjoen et al., 2017). PPIE was integral to the project through contributors who had lived experiences within the community. This contribution closely resembles those described in similar reports that highlight the benefits of engaging the community (Aries et al., 2021; Crowe et al., 2021). Working alongside PPIE advisors helped enhance the study by giving careful consideration to ensure transparency, ethical integrity, and inclusiveness. Their ‘lay perspectives’ helped enhance the research by complementing the technical perspectives of the researchers, a reflection that has been highlighted in similar experiences with participatory design (Biggane, Olsen, & Williamson, 2019). A good example was using popular slang and colloquial terms instead of medical terms and observing sensitivity to Malaysian cultural and social contexts.

The ADDIE design of instruction was deemed more suitable for this project due to its adaptability across a wide range of fields (Davis, 2013). The ADDIE design of instruction allowed researchers and PPIE advisors to define the entry point and refine or revisit any step at any time. The main challenge with any educational intervention designed for health promotion is making it “fit for consumption” (Singh et al., 2017). To overcome this challenge, it was imperative to make the scientific content understandable, usable, and acceptable to the end-users, which in our case were transgender women in Malaysia. Hence, the content was made bilingual, in both English and Bahasa Malaysia, through a back-and-forth translation process in both languages, ensuring that the message delivered has the same impact. Incorporating the local language into the health messages was deemed essential for its effective delivery and enhancing the connection with the audience. Since the translation and content creation were done in collaboration with PPIE in place, the educational intervention is believed to be persuasive and simultaneously improve trust (Bonevski et al., 2014; Clayman et al., 2010).

Research findings by the authors (Telang et al., 2025), as well as similar reports involving transgender women from other parts of the globe (Robbins et al., 2020; Sarwar et al., 2023) report routine engagement in unprotected oral sexual practices, often with multiple partners. The common finding in these reports is that the participants associated unprotected oral sexual activity with a low risk of acquiring STIs (Robbins et al., 2020; Sarwar et al., 2023; Telang et al., 2025). The focus of this educational intervention through the 3-week pilot was hence directed toward both STI prevention and oral health, intentionally combining these topics to highlight the risk of STI transmission through unprotected oral sexual practices. The pilot study was conducted with the aim of optimizing the intervention for a larger study rather than directly assessing behavioral change, as is typical of pilot studies that are not particularly powered to assess changes in outcomes (Lancaster, Dodd, & Williamson, 2004). Feasibility with social media-based research was measured based on participant recruitment and retention. Since the current pilot successfully retained all 30 participants within the group until the end of the study, it was considered feasible. However, 2 of the participants chose not to answer the post-intervention questionnaire, stating time constraints as the reason. This feedback was crucial in making the necessary changes to the questionnaire for a larger-scale version of the study. The measure of acceptability of the study relied on the open-ended feedback provided by the participants and their chances of recommending the intervention to other transgender women. The number of likes per post averaged only 5–6, which may not be an accurate method of assessing actual engagement, as participants may have been viewing the content but not visually expressing their interest through likes. The aim was to subtly facilitate engagement with the content rather than constantly demanding attention, which could have resulted in followers leaving the group due to information overload and fatigue (Ayari, Oueslati, & Ben Yahia, 2025; Farooq, Dahabiyeh, & Maier, 2023). Research literature describes disengagement with social media platforms and/or unfollowing groups as a complex, multifaceted phenomenon (Ernst & Schnauber-Stockmann, 2024; Farooq et al., 2023; Fu et al., 2020). The content itself can prompt disengagement; hence, staying relevant by avoiding repetitive and excessive information may be an important factor in sustaining the long-term interest of participants or followers (Ayari et al., 2025; Fu et al., 2020).

Most participants in the pilot study (82.2%) were satisfied with the educational content delivered through *Ms Radiance.* The open feedback suggested that the content was informative, useful, and easy to understand. Comments regarding better organization were planned to be addressed by posting similar posts and stories within the same week in order to maintain the continuity of the topic. Also, suggestions to adopt more accepted inclusive terminology, such as ‘transnita’, in Bahasa Malaysia were incorporated. This highlights the importance of using culturally sensitive language to ensure that the participants feel respected and valued. Soliciting such feedback from the community is an important guideline for conducting culturally sensitive research with transgender participants (Staples et al., 2018). Historically, cultural sensitivity incorporated into research has been shown to increase receptivity and acceptance, as well as influence target health behaviors (Resnicow et al., 1999). The suggestion for using more videos was considered, with a plan to involve transgender women community influencers narrating their lived experiences. It was interesting to note that the participants viewed the oral health information as often overlooked and informative; hence, more content on oral health and dental care would be added. Through the pilot feedback, we identified aspects that could enhance the scaled-up version of *Ms Radiance*. It was evident that the participants had the appetite for more information, and to keep the followers engaged, it was important to increase the frequency of posting every day or every alternate day. To make the scaled-up study reliable, a 3-month long study was planned which would include a larger and more diverse group of transgender women, in terms of ethnicity and geographical location in Malaysia. The effectiveness of the educational intervention was proposed to be evaluated through a scaled-up one-group pretest/post-test quasi-experimental study. The scaled-up version would be able to answer important questions on how to make the intervention sustainable on social media as well as its utilization at the community level for a wider audience.

## Strengths and limitations

The study displays many aspects that are recognized as strengths. Firstly, integrating PPIE at nearly all stages of the study design, as well as collaborating with the community to develop the educational intervention, was a notable achievement. Secondly, the endorsement of STIs and oral health research priority areas in vulnerable populations in this developing part of the world is also seen as a notable strength. Thirdly, the design and development of this one-of-a-kind bilingual, culturally sensitive, social media-based educational intervention is grounded in theory and serve as an example for future research in this field. Finally, the study also satisfies three of the United Nations Sustainable Development Goals (SGD3- Good health and well-being, SGD 5 gender equality, SGD 10 Reduce inequalities).

The study, however, had its fair share of limitations. The pilot was limited by a small number of participants, a non-randomized study design, and a short 3-week interval between pre- and post-intervention data collection. Moreover, the pilot study data only assessed the feasibility and acceptability of the educational intervention and were insufficient to report the actual impact on behavior change. The study focused on transgender women as a single entity, and diversity in participant characteristics such as ethnicity, education level, sexual orientation, and sexual behaviors on the research objectives were not individually reported. Expanding on this diversity could bring more clarity to a broader context. The lack of longitudinal clinical data in parallel to assess whether the educational intervention had a clinical impact was seen as a limitation. The measurement of participant engagement using Instagram analytics was not possible as the group was made private for participants’ privacy and security. The comments were also turned off for the same reason. The number of likes for a post or story was also small and was not deemed to be an accurate measure of assessing engagement during the pilot testing.

## Conclusion

This paper describes the participation of transgender community members in Malaysia in designing and developing an educational intervention delivered through social media. Dental service utilization and promotion of safe oral sexual practices relating to the transmission of STIs were targeted. The feasibility and acceptance of Instagram as an appropriate social media platform to deliver both sexual health and oral health education for transgender women in the Global South remain underexplored. This is a first-of-its-kind pilot to the best of our knowledge. PPIE in the study ensured cultural sensitivity and inclusiveness, which were successfully demonstrated through the visual appeal of the artwork and its influence on content creation. The ADDIE design facilitated the integration of instructional elements of health education into an organized yet simple and appealing delivery method. The strength of the educational intervention *Ms. Radiance* lies in the ability to deliver sensitive yet important health education for transgender women in an easy-to-understand and accessible format.

## Supplementary Material

Supplementary file GRIPP2SF Telang et al Ms Radiance.docx

## Data Availability

Due to the sensitive nature of the data collected in this study, it cannot be made publicly available. The data involves personal and confidential information, and sharing it could compromise participant privacy. However, specific aspects of the data may be made available upon reasonable request to the corresponding author, subject to approval by the ethics committee and in compliance with data protection regulations.
