# Supplementary material for: Ms Radiance: A community-led social media mHealth pilot for transgender women
Source: Int J Transgend Health. 2025 May 13;27(5):2309–25. doi: 10.1080/26895269.2025.2503224 (PMC13618165; doi:10.1080/26895269.2025.2503224)
Supplement: Supplementary file 1 [file WIJT_A_2503224_SM4349.docx]

**Guidance for Reporting Involvement of Patients and the Public (GRIPP-2) Short form Checklist**

| **Section and topic** | **Item** | **Reported on page No** |
| --- | --- | --- |
| Aim | The aim of this narrative is to describe the design and development of an educational intervention to improve oral health care utilization and promote safe oral sexual practices in relation to transmission of STIs for transgender women in Malaysia. PPIE was integral to the project to ensure participatory design and development of a bilingual, peer-reviewed and culturally sensitive social media based educational intervention. End-user experiences, feasibility and acceptability of the intervention were assessed through feedback obtained from a pilot study. | Page 6 |
| Methods  and results | Collaboration with transgender community to gain trust, collaboration between the principal author (LAT, a cisgender woman) and the research assistant (HSD, a transgender woman activist) expanded to include local transgender artists and other community members, ultimately forming a PPIE advisors’ team.  PPIE played a key role in the conceptualization and design of the study.  The involvement of the transgender community members was crucial to ensure the cultural sensitivity of the educational intervention.  Formative analysis of the topics was conducted during the FGD with a mixed group of PPIE advisors.  Participants were recruited through word of mouth and snowball sampling assisted by PPIE advisors through their community networks and from the pool of people who were involved in the initial exploratory research. | Pages 7,8 & 9 |
| Methods and results | Positive outcomes of PPIE in the research project-   - **Analysis:** A focus group discussion (FGD) was conducted with PPIE advisors, who provided key insights into the educational intervention. These advisors were carefully selected from a pool of individuals who had lived experiences and/or were working in the field of transgender healthcare. - **Community-reviewed educational content:** Keeping in mind that the content must be culturally sensitive and tailored to the needs of transgender women in Malaysia, it was essential that the content undergo community review. The content was made bilingual, in English and Bahasa Malaysia. Back-and-forth translation into both languages, incorporating local slang words, was used to make the message more relatable to the audience. This was done with the help of local activists from the transgender community. The name “Ms Radiance” was also chosen by the PPIE advisors as it reflects happiness, beauty, and good health seen on one’s face (Cambridge Dictionary (n.d.), 2024, ). Inputs from the PPIE advisors and consultations with experts in the field further strengthened the content. - **The role of the artists** was to simplify the content and convey the message through visual art. By incorporating positive transgender representation into the artwork, they helped make the content both appealing and ethically reflective (Caldwell, 2022) simultaneously. (Fig. 1 Sample posts and stories from Ms Radiance). Fictitious animated transgender characters in the iconic pink and blue colors of the transgender flag were hand-drawn and then digitized. The art form of Cubism was also used to depict the artist's impression of these characters (Ganteführer-Trier, 2004). - **Participants and Recruitment:** Participants were recruited through word of mouth and snowball sampling, assisted by PPIE advisors who leveraged their community networks and drew from the pool of individuals involved in the initial exploratory research. - The use of more accepted colloquial term for transgender women “Transnita” (Bahasa Malaysia) was suggested. | Page 8  Pages 9-10  Pages 10-11  Page 12  Page 25 |

|  | Limitation of the outcome of PPIE in the research project. PPIE was not seen as a limitation in this project, yet there were aspects of PPIE that could have been improved such as:   - Time management - Wider engagement with participants from other parts of Malaysia rather than only those who are within the friend's networks. - Designated funding for PPIE |  |
| --- | --- | --- |
| Discussion and conclusions | - The role of PPIE at various steps of the development process has also been credited. The pilot study with a group of transgender women in Northern Malaysia describes the feasibility and acceptance of Ms Radiance on Instagram. - The artists who contributed emphasized the creativity and visual appeal that resonated with the end user, a focus also reflected in comparable smartphone-based educational interventions (Hill & Johnson, 2020; Kuhns et al., 2021; Sun et al., 2020). The participants valued the emphasis on Ms Radiance being an exclusive social media group for transgender women. - PPIE in this research project provided us with the potential to improve the awareness and relevance of the research within the given timeframe and resources. - The benefits of community partnership facilitated access, participant recruitment as well as interpretation and contextualization of the results (Bouman, 2018; T’Sjoen et al., 2017). PPIE was integral to the project through contributors who had lived experiences within the community. - Working alongside PPIE advisors helped enhance the study by giving careful consideration to ensure transparency, ethical integrity, and inclusiveness. Their ‘lay perspectives’ helped enhance the research by complementing the technical perspectives of the researchers, a reflection that has been highlighted in similar experiences with participatory design (Biggane et al., 2019). | Page 25-27 |
| - Reflections /critical perspective | Good practice of including PPIE authors in the research   - Integrating PPIE at nearly all stages of the study design, as well as collaborating with the community to develop the educational intervention, was a notable achievement. - The artwork was created by the artists from the transgender community.   Reflexivity and positionality:  The authors of this article include cis-gender and transgender individuals. A reflexive approach was maintained throughout the study period through respectful communication, ensuring confidentiality, valuing each other’s feedback, and transparent reporting. Transgender women were directly involved in participant recruitment, data collection, analysis, and sharing of findings. Three of the four authors are from the Global South, with two of them being Malaysian. One of the authors is a transgender woman who received training in PPIE research methodology and the remaining authors have experience working with gender-diverse populations. All authors involved in the interpretation of data acknowledge their identities and experiences may influence the interpretation and are committed to ensuring a reflexive approach free of potential biases. | Page 6 |
